# Impaired Early-Response Inhibition in Overweight Females with and without Binge Eating Disorder

**DOI:** 10.1371/journal.pone.0133534

**Published:** 2015-07-22

**Authors:** Jennifer Svaldi, Eva Naumann, Stefanie Biehl, Florian Schmitz

**Affiliations:** 1 Department of Clinical Psychology and Psychotherapy, University of Tübingen, Tübingen, Germany; 2 Department of Clinical Psychology and Psychotherapy, University of Freiburg, Freiburg, Germany; 3 Institute of Psychology and Pedagogy, Ulm University, Ulm, Germany; University Hospital of Bellvitge-IDIBELL; CIBER Fisiopatología Obesidad y Nutrición (CIBERObn), Instituto Salud Carlos III; Department of Clinical Sciences, School of Medicine, University of Barcelona, SPAIN

## Abstract

**Objective:**

Several studies report increased reward sensitivity towards food in overweight individuals. By contrast, data is inconclusive with respect to response inhibition in overweight individuals without binge eating disorder (BED). Hence, the latter was addressed in the present study in a group of overweight/obese females with and without BED and a normal-weight control group without eating disorders.

**Method:**

A group of women with BED (*n* = 29), a group of overweight women without BED (*n* = 33) and normal-weight females (*n* = 30) participated in a pictorial priming paradigm, with food items (relevant primes) and office utensils (neutral primes) and color blobs (neutral primes) as stimuli. Increased response priming effects (i.e. priming with switches between stimulus categories) were taken as indicators of deficient behavioral inhibition.

**Results:**

Priming effects for neutral primes were moderate and comparable across all groups. However, primes associated with the food task set lead to increased priming effects in both overweight groups. But, effects were comparable for overweight/obese participants with and without BED.

**Discussion:**

Results suggest that early response inhibition in the context of food is impaired in overweight individuals compared to normal-weight individuals.

## Introduction

Data from the recently published Global Burden of Disease study [1] shows a dramatic increase of overweight and obesity combined of 27.5% for adults and 47.1% for children over the past thirty years. Overweight and obesity are associated with substantial medical [2–4], psychological [5, 6] and psychosocial sequelae [7–10], a reduced health-related quality of life [11] and an increased mortality risk [12]. Particularly due to the associated comorbid physical and mental diseases, obesity causes enormous costs for the medical systems [13–15]. Both psychological interventions [16], as well as anti-obesity drugs, meal replacements and high-protein diets [17] were shown to be associated with a significant weight reduction. However, most individuals are still overweight or obese after extensive treatment [18], and stabilization of weight loss following treatment is difficult and often fails [19, 20]. Thus, there is a great need for research identifying maintaining factors of overweight and obesity in order to enhance the development of effective treatments [21, 22].

One of the issues frequently addressed in obesity research is the obesogenic food environment, which on the one hand promotes sedentary behavior, on the other hand fosters high energy intake [23] due to the omnipresence of palatable food. However, not all individuals are equally susceptible to the constant temptations of palatable food, and some individuals are better able than others to decide themselves against the hedonic pleasure of eating in order to avoid the negative long-term consequences of increased food-intake.

Evidence suggests that impulsivity may play a prominent role with regard to increased food intake. First of all, a number of self-report studies show that obese individuals describe themselves as more impulsive than normal weight individuals [24, 25]. Furthermore, obesity goes along with an increased prevalence of impulsive behaviors such as substance abuse [26], attention-deficit hyperactivity disorder [27] and other impulsive behaviors (e.g., compulsive buying; [28]). Beyond self-report, obese females were shown to be characterized by greater delay discounting [29, 30], an index of diminished self-control, when compared to normal weight females. In addition, in a gambling task they showed a preference for immediate rewards (i.e., gains) at the cost of long-term consequences (i.e., larger losses), which was also considered indicative of impulsive decision making [30, 31].

However, impulsive behavior may result as a consequence of multiple factors. In this respect, Gray’s revised reinforcement sensitivity theory [32–34] may serve as a conceptual framework, positing three biologically grounded systems: The behavioral activation system (BAS) governs hedonic approach towards rewarding stimuli, whereas the fight-flight-freeze system (FFFS) responds to punishment-associated stimuli. Finally, the behavioral inhibition system (BIS) is responsible for detecting response conflict and for inhibiting behavior automatically triggered by the other motivational systems. Generally, it is predicted that high BAS and low BIS are susceptibility factors for impulsive behavior. Applied to binge eating disorder (BED) and obesity, the interplay of a high behavioral activation towards rewarding food items and an impaired behavioral inhibition can be predicted to contribute to binging (and over-eating). In support of the two-systems perspective, Dawe and Loxton [35] reviewed existing literature on self-report impulsivity assessment and identified two major factors, namely “reward-sensitivity” and “rash-spontaneous impulsiveness”. The first clearly corresponds with Gray’s BAS and the latter may result in case of impaired BIS. Importantly, both factors were shown to be related with BED. Further evidence of the role of these systems in eating disorders was summarized in a recent review paper on the neural responses to food cues in individuals with eating disorders and obese individuals relative to the general population [36]. Two brain circuits were identified that distinguish between these groups and controls: The first comprises limbic and paralimbic areas associated with salience and reward, and the second comprising prefrontal areas that are assumed to be required for cognitive control. In the following, core findings related to increased food-related reward sensitivity and impaired inhibition will be summarized for overweight/obesity and BED, taking into consideration different lines of research (comprising behavioral data, functional imaging, and evoked potentials).

With respect to the reward-related approach system, several fMRI studies have shown that food is much more rewarding for overweight individuals than for normal weight individuals (see [36] for an overview). For example, a number of studies found an increased activation in reward-specific regions in overweight and obese compared to normal weight individuals when confronted with (high-caloric) food stimuli [37–39] or anticipated food and food intake [40]. Research using the visual probe task as a behavioral indicator of attention allocation has shown that obese participants revealed larger response-time based stimulus engagement effects than controls [41], which can be interpreted as an indicator of attention approach.

Also for BED, evidence supports that food-related stimuli are perceived as particularly rewarding. For instance, a functional imaging study with BED patients has shown that food pictures elicit elevated activation in the medial orbito-frontal cortex in participants with BED relative to controls [42]. Converging evidence comes from an EEG study using long-latency potentials (LLPs) as an indicator of motivational properties, and showing that BED patients displayed larger LLPs for high-caloric food pictures compared to overweight controls [43]. Additionally, food stimuli appear to generally increase response preparedness in BED, as suggested in a functional imaging study in which food stimuli were shown to elicit greater activity in right pre-motor areas in BED than controls [44]. Similarly an EEG study investigated beta wave activity as an indicator of motor preparedness. They found beta activity to be generally increased when food stimuli were presented relative to neutral stimuli, but this difference was more pronounced in participants with BED relative to controls [45]. Finally, also behavioral data collected with the spatial cueing task support that participants with BED orient their attention faster to food stimuli than weight matched controls [46].

With respect to response inhibition, the data is much more inconclusive. So far, one study used the go/no-go task with food and neutral words and found no significant differences between overweight and normal weight men and women [47]. Likewise, by means of a semantic priming paradigm, another study found no significant association between high-calorie food words and disinhibition words in a sample of overweight and obese females compared to a normal weight control group [48]. Effects in the semantic priming paradigm are considered to indicate spreading activation in an associative knowledge network (e.g., [49]). However, disinhibition was not measured at the performance level in this study. Finally, in a recently published eye-tracking study overweight individuals did not display more difficulties voluntarily suppressing first saccades to peripheral food cues in an antisaccade paradigm [50]. By contrast, several fMRI studies and studies using behavioral measures found evidence of inhibitory deficits in overweight individuals compared to normal weight controls (see [36] for an overview). As such, one MRI study found a greater activation in areas of the prefrontal cortex associated with inhibition in satiated obese compared to satiated normal weight men [51]. Comparably, one fMRI study found a higher activation of prefrontal cortex regions in response to food pictures (but not in response to control pictures) in obese compared to normal weight (male and female) children [52]. Another study reported a significant negative correlation between body mass index (BMI) and activation of frontal inhibitory regions in adolescent girls ranging from lean to obese [53]. Of note, this study adopted a food-related go/no-go task and also found behavioral response inhibition to negatively correlate with BMI. In contrast to Loeber et al. [47], who used food-related and neutral words, this study used high and low caloric food pictures, which might be more salient. This is also in line with a recent study [54] in which a higher BMI was found to be associated with decreased inhibitory control in a food-related Stop-Signal Task (SST), but not in a SST with neutral stimuli. Hence, overweight individuals may display inhibitory response-deficits mainly in the context of food. General impairments in cognitive functioning as a function of obesity were also found in other studies using multivariate cognitive assessment batteries [55–57].

Findings are less conclusive whether individuals with BED have inhibitory deficits over and above overweight/obese participants. For instance, in one study using a modified affective shifting and go/no-go task [58], all obese participants committed more errors than normal-weight controls. However, obese participants with BED made significantly more errors than those without BED. In another study, a SST with food and neutral stimuli was administered to obese participants with and without BED [59]. Participants with BED generally needed more time to cancel an ongoing response relative to controls, additionally, inhibition was particularly impaired when the response was elicited by food stimuli. However, when using a SST with only neutral stimuli no difference was found between a BED group and weight-matched controls [60]. Similarly, in one sample of “morbidly obese participants” [55], no differences were found between participants with and without BED in a multivariate cognitive assessment battery. The latter study exemplifies as well that inclusion criteria of the control group are not trivial.

One factor that complicates the findings on behavioral inhibition is the fact that all but two studies [50, 58] did not control for the presence of binge eating disorder (BED). As individuals with BED were previously shown to be characterized by rash-spontaneous behavior compared to overweight individuals without BED [45, 58, 59], more studies assessing rash spontaneous behavior in overweight and obese individuals without BED are needed [61].

Second, different factors of inhibitory control were shown to be separable [62, 63]. The antisaccade paradigm used in pure overweight and obese individuals in the Schag, Teufel, et al. [50] study assesses inhibition of oculomotor orienting reactions, while the paradigms used in the Mobbs, et al. [58] and the Batterink, et al. [53] study reflect response inhibition in settings with arbitrary S-R relations. Of note, even though all these paradigms are classically considered as indicators of behavioral inhibition, task performance in different laboratory paradigms is usually hardly correlated [62, 63], either indicating task-specificity or separability of the required inhibitory functions. Hence, overweight/obese individuals without BED may not experience a particular difficulty in oculomotor inhibition [50]. By contrast, they appear to experience a difficulty in inhibiting an overt response [53, 58].

To conclude, across different lines of research, previous studies have offered evidence that obese/overweight individuals and individuals with BED have elevated food-related rewards responsiveness [35, 36]. Concerning inhibitory control, findings are less conclusive. However, inhibition seems to be impaired in overweigh/obese participants, and some evidence has been found that BED-specific impairments exist. The latter may depend on whether responses were elicited in the context of relevant and arousing stimuli. However, two issues remain unsatisfactory and need to be addressed in future research: First, in many studies, the specific cognitive mechanisms are largely ignored or experimental tasks are interpreted as interchangeable indicators of broad, multifacetted constructs–which they may not be. Second, in many studies overweight/ obesity and a BED diagnosis are confounded, thereby complicating the interpretation of effects.

Consider first specific mechanisms and the use of experimental tasks. While an interplay of a motivational reward system and an inhibitory control system is widely accepted as a heuristic in impulsivity research (see [64] for a discussion), the specific mechanisms how and when “impulsive” and controlled processes interact still deserves investigation. Current taxonomies conceptualize inhibition as a multifacetted construct, governed by overlapping but separable control functions [62, 65]. Specifically, these inhibition and interference control functions were postulated to operate at different stages of information processing, i.e. stimulus interference control, cognitive interference control, interference control at response selection, and response cancellation [65, 66], and they were shown to be characterized by partly overlapping as well as specific neural circuits [67]. This has a number of implications: There is definitely no one-to-one mapping of tasks and functions. However, different laboratory tasks may differentially require separable inhibitory functions. Consequently the tasks are not interchangeable indicators of the same inhibition ability. This could also account for some of the inconsistencies found in previous research. Further, theoretically motivated research should systematically seek to investigate those functions not previously tested to complete the picture. So far, previous studies on inhibitory deficits in overweight/obesity and BED have worked with a limited number of well-established tasks that primarily focus on late-stage inhibition. In particular, the SST (e.g., [54, 59, 60]) indexes late-stage cancellation of an already elicited response. The go/no-go task (e.g., [47, 53, 58]) may tap a somewhat earlier stage of action withholding, but response cancellation may also contribute to task performance. The antisaccade task [50] differs from the previous tasks as it requires the suppression of an oculomotor orienting reflex (whereas the other tasks use arbitrary S-R rules and afford overt motor responses). However, the antisaccade task has been considered an indicator of classical behavioral inhibition together with stop-signal and go/no-go task [65, 66]. Conversely, the earlier stage of response selection has not been specifically addressed in previous overweigh/obesity or BED research.

Consider next the confounding of overweight/obesity with BED. In previous research addressing overweight/obesity, in none but two studies [50, 58] the presence of binge eating disorder (BED) was explicitly controlled for. This provokes the question whether some of the “impulsivity” observed in obese samples may actually be driven by a sub-sample of participants with BED. In fact, inhibitory functions were shown to be impaired in individuals with BED compared to overweight individuals without BED [45, 58, 59]. Consequently, more studies assessing rash spontaneous behavior in overweight and obese individuals without BED were demanded [61]. Conversely, virtually all individuals with BED are also overweight or obese and were consequently compared with overweight/obese controls in previous research (e.g., [55, 60]). The latter is a straight-forward contrast of the unique effect of BED. However, this leaves unresolved how large a possible effect is in comparison with the difference of both obese groups relative to normal-weight controls.

The aim of the present study was two-fold: The first purpose of this study was to investigate inhibition at the early phase of response selection. To this end, a response window priming task [68, 69] was used which was recommended as a highly specific marker of this control function [66]. The task was modified to comprise food and neutral primes in order to test their possibly differential interference effects. The second purpose of this study was to tear apart possible effects of overweight/obesity from that of BED. To this end, three groups were investigated: (a) overweight and obese individuals with BED, (b) overweight and obese individuals without BED, and (c) normal weight individuals without lifetime eating disorder.

Taking into account previous findings that inhibition was more consistently impaired in overweight/obesity and BED in the context of food related stimuli, we set up the following hypotheses for the current study: (1) All groups experience comparable interference at response selection as long as the presented stimuli are neutral. (2) However, when food-related stimuli are presented, interference effects are increased more strongly in overweight/obese individuals than in normal weight controls. (3) Additonally, overweight/obese participants with BED may experience stronger interference than overweight/obese participants without BED. (4) The magnitude of early response interference is related to eating pathology.

## Materials and Methods

### Participants

The present study has been approved by the ethics committee of the University of Freiburg (253/10). Written informed consent was obtained from all participants. They were remunerated 20€ for study participation.

Participants were recruited by means of announcements in local newspapers, television, and radio. Data collection was completed from 2012/02 to 2014/02. Inclusion criterion for the BED group was the presence of BED according to DSM-5 criteria (American Psychiatric Association, APA [70]). A total of 92 women participated in the study, 29 with a diagnosis of binge eating disorder, 33 overweight without BED, and 30 normal weight controls. BED patients had a BMI range of 26.4 to 43.9, thereby, all of them were either overweight (*n* = 3; BMI < = 29.9) or obese (*n* = 27; BMI > = 30.0). Consequently, this group will be referred to as OW/OB+BED in the following. Six of the participants were in psychotherapeutic treatment at study entry. Inclusion criterion for the overweight/obese control group (OW/OB-BED, in the following) was a BMI (weight/height^2^) ≥ 25 (and an actual range from 26.4 to 43.9; *n* = 12 overweight, *n* = 21 obese) in the absence of a lifetime eating disorder. Participants in the normal-weight (NW) group were included if their BMI was between 18.5 and 24.9 with an absence of a lifetime eating disorder. Only adult woman were included in this study. Exclusion criteria for all groups were the presence of current substance abuse or addiction (except sustained full remission), bipolar disorder, current or past psychosis, schizophrenia and current severe suicidal ideation. Socio-demographic details can be obtained from Table 1. A oneway analysis of variance (ANOVA) conducted on BMI confirmed a significant main effect of Group (*F[*2, 89] = 92.418, *p* < 0.001, *η* ^2^ = 0.675). The BMI in the NW group was significantly lower than in both obese groups (both *p*s < 0.001), but comparable in the latter groups (*p* = 0.84). There were no other significant group differences on socio-demographic variables with regard to age (*F*[2, 89] = 1.004, *ns*, *η* ^2^ = 0.022), marital status and educational level (all *χ* ^2^s<8.076, all *p*s>0.426).

**Table 1:** Sociodemographics and overall psychopathology presented separately for participants in the normal weight (NW), the overweight/obese without BED (OW/OB-BED), and in the overweight/obese group with binge eating disorder (OW/OB+BED).

		*Mean (Standard Deviation)*			
		NW (*n* = 30)	OW/OB-BED (*n* = 33)	OW/OB+BED (*n* = 29)	Post-hoc Tests
Age		42.67 (15.07)	41.97 (14.34)	46.83 (13.63)	NW = OW/OB-BED = OW/OB+BED
BMI		22.00 (1.79)	32.98 (1.79)	34.73 (4.10)	NW<OW/OB-BED = OW/OB+BED
Marital status					NW = OW/OB-BED = OW/OB+BED
	single	9	7	6	
	partnership	10	10	5	
	married, cohabitate	3	10	7	
	married, not cohabitate	2	2	2	
	divorced/widowed	6	4	9	
Educational level					NW = OW/OB-BED = OW/OB+BED
	low	12	13	13	
	high	18	20	16	
EDE-Q_GS_		0.92 (0.96)	1.23 (0.82)	3.65 (0.75)	NW = OW/OB-BED < OW/OB+BED
EDE-Q_RE_		0.81 (1.22)	0.79 (0.97)	2.41 (1.22)	NW = OW/OB-BED < OW/OB+BED
EDE-Q_EC_		0.27 (0.31)	0.22 (0.31)	2.88 (0.92)	NW = OW/OB-BED < OW/OB+BED
EDE-Q_WC_		0.93 (0.94)	1.48 (1.02)	4.12 (1.06)	NW<OW/OB-BED < OW/OB+BED
EDE-Q_SC_		1.29 (1.36)	1.82 (1.30)	4.34 (0.89)	NW = OW/OB-BED < OW/OB+BED
BDI-II		6.57 (6.95)	4.97 (5.02)	16.76 (10.09)	NW = OW/OB-BED < OW/OB+BED

BMI = body mass index (weight/height^2^); EDE-Q = Eating Disorder Examination Questionnaire; GS = global score; RE = restraint subscale; EC = eating concerns subscale; WC = weight concerns subscale; SC = shape concerns subscale; BDI-II = Beck Depression Inventory.

The OW/OB+BED group had a mean of 3.66 (*SD* = 1.57; range 1–7) binge days per week over the past six months prior to testing. As expected, there were significant main effects of group with regard to severity of eating pathology and depression (all *F*s > 19.719, all *p*s < 0.001, all *η* ^*2*^s = 0.307): Compared to the OW/OB-BED and NW groups, participants in the OW/OB+BED group scored significantly higher on scales assessing severity of eating pathology and overall psychopathology (all *p*s < 0.001). Furthermore, the OW/OB-BED group reported higher weight concerns than the NW group (*p* = 0.032). There were no other significant differences between the OW and NW groups on any other variables related to eating disorder psychopathology or severity of depression (all *p*s > 0.083).

### Pictorial response priming task

**Fig 1:**
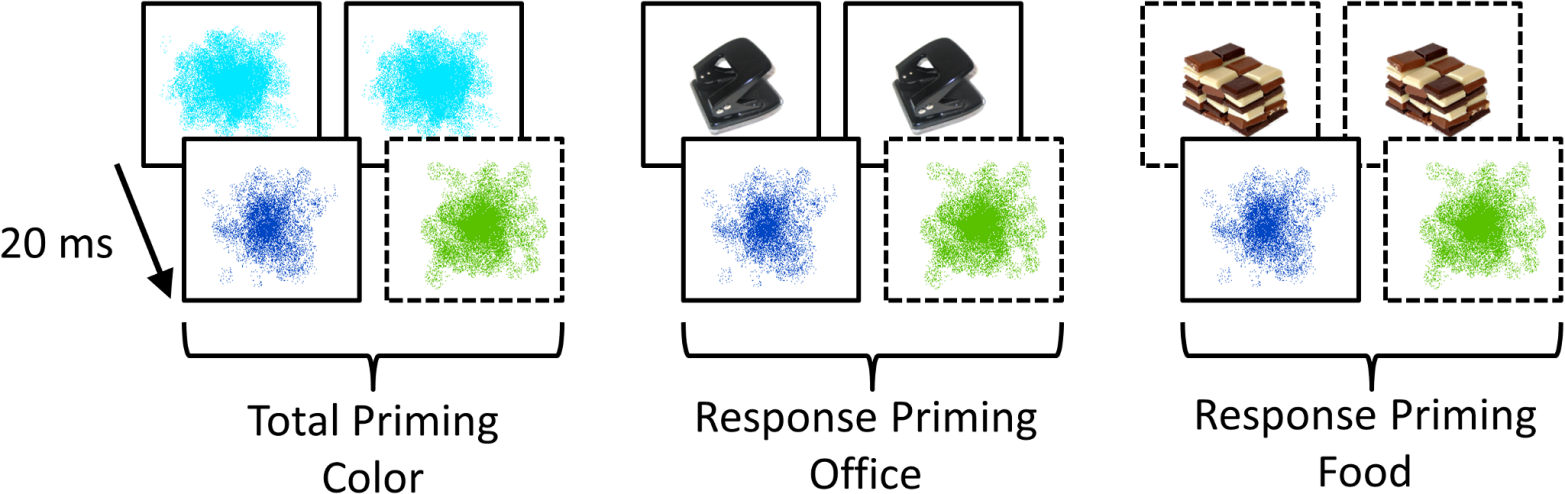
Schematic presentation of the adopted pictorial response-priming task.

In each trial, two stimuli were presented in rapid succession, i.e. the prime and the probe stimulus (see Fig 1). Participants were instructed to ignore the first stimulus and to classify the second according to two response rules: Photographs of common stimuli had to be classified as office utensils or food items, whereas color blobs had to be identified as blue or green. Color categories were chosen because they neither appeared to be semantically nor affectively associated with office utensils or food items. There were eight stimuli of each category. Office utensils included photographs of, e.g., a hole puncher, a pocket calendar, and an eraser. Food stimuli included photographs of, e.g., chocolate, fries, and sandwiches. The color stimuli had different color tones, additionally, each paint-brush type color blob had a different shape. The photographs of food and office items have been previously employed in an EEG study with BED patients [43], and the color blobs were the same as those in the response priming paradigm employed in the original study [66]. All stimuli had a size of 230 px (w) x 170 px (h) and were presented in the center of a light gray screen (RGB = 220, 220, 220) surrounded by a thin black rectangle that was visible throughout the block to help focus attention. All stimuli could appear as primes or probes in a pseudo-random order, balancing all sequences of stimulus categories. All combinations of response-mappings were counterbalanced across participants. The prime-probe interval was fixed at 20 ms.

The prime (rear row) is followed by a probe (front row) in short succession (20 ms) in all trials. As all stimuli could appear as probes, they were associated with a left or right response in line with the relevant task set and previously acquired S-R associations. In this schematic, blue and green color blobs needed to be classified by pressing a left and right response, respectively. Analogously, office utensils and food items had to be classified by left and right button presses, respectively. For clarity, rectangles displayed with continuous and interleaved lines indicate that the stimulus is associated with a left and right response, respectively. (In reality, all rectangles shown were identical, and all combinations of response mappings of the stimuli were counterbalanced across participants.) Although participants are instructed to ignore primes, the prime may automatically elicit an associated response that can interfere with response selection when the probe is classified in line with task instructions. Consequently, there may be facilitation in case of response-congruent prime-probe pairs and response interference in case of response-incongruent prime-probe pairs. The priming effect denotes the difference in response times and errors in response-incongruent vs. response-congruent prime-probe pairs. Large priming effects indicate that the prime stimulus strongly interferes with probe-based response selection, and have been considered an indicator of insufficient response inhibition / interference control at the early stage of response selection.

The session started with three learning blocks of 16 trials each: In the first, only office utensils and food stimuli could appear as probes and had to be classified accordingly. In the second, only blue and green color blobs were shown as probes. In the third block, both tasks were combined. Next, two training blocks of 66 trials each were presented in which a response window was introduced. For the duration of the response window, the color of the rectangle surrounding the stimuli changed from black to yellow (RGB = 250, 250, 50), and returned to black again after the end of the response window. If participants managed to give their responses within the response window, the stimuli changed their color to white, but their contour remained in place until the end of the trial. The trial ended 300 ms after giving the response, irrespectively of whether it was given in the window or not. The duration of the response window was 140 ms, and its center was originally set at 500 ms after probe onset. The response window was individually adjusted after each block by shifting the window 30 ms earlier or later if the error rates exceeded the range of 20–45%. Four different inter-trial-intervals (360 ms, 420 ms, 480 ms, and 540 ms) were used to make it more difficult for the participants to prepare for the onset of the next prime stimulus, e.g. by dampening attention thereby escaping distraction. After each block, a performance feedback was given, including the proportion of correct responses, the average response time, and how many responses were given within the response window. After the training phase, there were 12 test blocks of 64 trials each (plus two warm-up trials) that were used for the analyses. The experiment was controlled by a compiled C++ program using the SDL libraries for stimulus presentation and response collection.

We investigated priming effects in trials with neutral color blobs as probes. As the same stimuli are presented and the classification affordance is the same in all these trials, systematic effects of the preceding stimulus can be interpreted as priming effects (see Fig 1). If the prime stimulus is a color blob, priming effects can occur in different phases: e.g., at encoding, at lexical access, and also at response selection. Consequently, the difference between response-incongruent prime-probe pairs and response-congruent prime-probe pairs constitutes the total priming effect. Differently in trials with office utensils or food items as primes, there is a category switch between primes and probes. Consequently, the only source of a possible priming effect is response facilitation or interference, depending on the prime-probe response-congruency. Priming effects can be reflected in response times and error rates, so both will be reported. Additionally, a compound score was computed by averaging standardized response-time and error priming effects [63], integrating both sources of information into a common metric.

### Questionnaires and interviews

The following questionnaires were administered online two to five days prior to testing. (1) The Eating Disorder Examination Questionnaire (EDE-Q) [71, 72] is a self-report measure that assesses the presence and severity of eating pathology. It consists of a global score and four subscales (restraint scale, eating concern scale, weight concern scale and shape concern scale) with high internal consistency (0.85 ≤ Cronbach’s *α* ≤ 0.93, depending on the scale) and stability (0.82 ≤ *r*_*tt*_ ≤ 0.88, depending on the scale) [71]. (2) The Beck Depression Inventory-II (BDI-II) [73, 74] assesses severity of depression over the last two weeks, and several studies have confirmed the BDI-II’s high internal consistency (*α* = 0.84), test-retest reliability (*r*_*tt*_ = 0.75) and discriminant validity [75]. Lifetime eating disorders were assessed with the Eating Disorder Examination (EDE) [76, 77]. All other diagnoses were determined by means of the Structured Clinical Interview (SCID) for DSM-IV Axis I [78, 79].

### Procedure and data analyses

The diagnostic interview and the experimental part of this study were administered in two independent sessions. The latter took place either at 10 am or at 2 pm. Participants were instructed to eat a regular sized breakfast/lunch the day of testing, but to abstain from eating 2 h prior to the testing session. Compliance was checked verbally by the laboratory assistant at study entry. Then participants were administered the behavioral paradigm, after which they were discharged from the study.

Possible priming effects were tested in response times, errors and their compound scores (see [66]). The hypothesized pattern across prime categories and experimental groups was investigated simultaneously using mixed analyses of variance (ANOVA) using the statistical software R [80]. Prime category (color blobs vs. office items vs. food items) was specified as a within participants factor and group (OW/OB+BED vs. OW/OB-BED vs. NW) as a between participants factor. In case of a significant interaction, the pattern of predicted present and absent effects was tested with planned contrasts and all results are fully reported without adjustment of the alpha or beta level (see [81]).

## Results

### Data pre-processing and preliminary analyses

Extreme values were excluded according to an individually adjusted RT criterion [82]. Responses were excluded if they were longer than 1.5 interquartile ranges on top of the 75^th^ percentile of the distribution of correct response times and if they were faster than 1.5 interquartile ranges below the 25^th^ percentile or below 200 ms.

Following the rationale of the horse-race model that is underlying the stop-signal paradigm [83, 84], response interference in the priming task will be the stronger the faster the prime elicits a response relative to probe presentation. Consequently, we checked comparability of response times in the four categories. An ANOVA across response times with probe category (green vs. blue vs. office utensils vs. food items) as a within participants factor and group (OW/OB+BED vs. OW/OB-BED vs. NW) as a between participants factor revealed only a main effect of the category factor (*F* [3, 267] = 73.98, *p* < .001, *η*_*p*_^2^ = .45). Neither the main effect of group (*F* [2, 89] = 1.11, *p* = .33, *η*_*p*_^2^ = .02) nor the interaction of both factors (*F* [6, 267] = 1.06, *p* = .39, *η*_*p*_^2^ = .02) was significant. Post-hoc analyses revealed that response times for the green color blobs (*M* = 588, *SD* = 137) and for the blue color blobs (*M* = 590, *SD* = 134) were comparable (*p* = .29), but color stimuli were generally faster classified than office utensils (*M* = 611, *SD* = 138) and food items (*M* = 618, *SD* = 142) (all *p* < .001). Additionally, response times were faster for office items than for food items (*p* < .01). In line with the horse-race model, this suggests that larger priming effects could be predicted for trials with color stimuli as primes, followed by office utensils and finally food items. This implies that the magnitude of the priming effect for different prime types could reflect difficulty (onset of the prime-elicited response) additionally to a possible moderation by stimulus relevance (food vs. neutral) of the trial category. However, a group comparison within trial type seems to be more easily warranted given the absence of a group main or interaction effect.

### Priming effects

**Fig 2:**
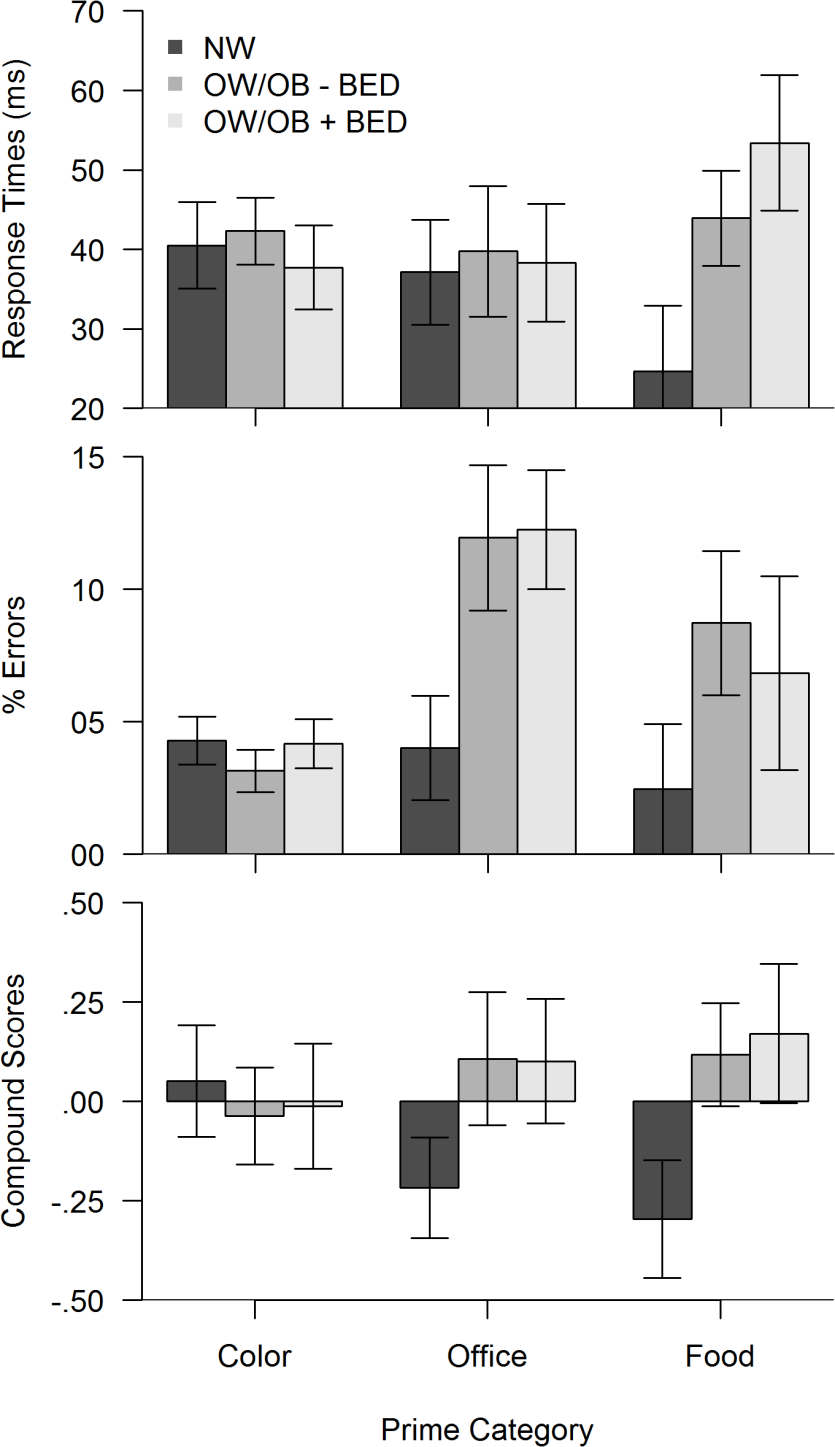
Priming effects in color prime, office prime and food prime trials.

All investigated priming effects are displayed in Fig 2, in the upper part, response-time based effects, in the middle part error-based effects, and in the lower part priming effects in the compound score metric. On the left are the total priming effects in trials with color primes for comparison purposes. In the middle and right, the specific response priming effects for office utensils and for food items as primes are displayed. The specific patterns were investigated next within each measure with ANOVAs with prime type (color vs. office utensil vs. food item) as a within participants factor and group (OW/OB+BED vs. OW/OB-BED vs. NW) as a between participants factor.

Large priming effects indicate insufficient response inhibition / interference control at early response selection, and can be reflected in response times (upper display) and in commission error rates (middle display). In order to integrate both bits of information into a common metric, compound scores (lower display) were computed by standardizing and averaging RT and error scores. Due to standardization, the mean of the compound scores is zero, whereas negative and positive compound scores indicate below average and above average priming effects, respectively. The bars depict group means (with standard errors) for the normal weight group (NW), the overweight/obese group without BED (OW/OB-BED), and in the overweight/obese group with binge eating disorder (OW/OB+BED).

In the ANOVA across response time effects, there was neither a main effect of prime type (*F* [2, 178] = 0.14, *p* = .87, *η*_*p*_^2^ < .01), nor a main effect of the group factor (F [2, 89] = 0.89, *p* = .41, *η*_*p*_^2^ = .02), but an interaction of both factors (*F* [4, 178] = 2.45, *p* < .05, *η*_*p*_^2^ = .05). Post-hoc tests revealed that the interaction was driven by the absence of any group differences in the color prime and office prime conditions (all *p* >.50). Confirming predictions, only in the food prime condition, both overweight groups had larger priming effects than the normal weight group (OW/OB-BED: *p* < .05, one sided, and OW/OB+BED: *p* < .05, respectively), but the priming effects in both obese groups were of comparable magnitude (*p* = .36).

The ANOVA across error-based effects, revealed a main effect of prime type (*F* [2, 178] = 7.47, *p* < .001, *η*_*p*_^2^ < .08), indicating generally larger priming effects for office and food primes compared to the color primes. There was no main effect of the group factor (*F* [2, 89] = 2.02, *p* = .14, *η*_*p*_^2^ = .04), and the interaction of both factors indicating larger priming effects in trials with office and food primes in both overweight groups relative to the normal weight group missed significance (F [4, 178] = 2.31, *p* = .06, *η*_*p*_^2^ = .05). Priming effects in office and food prime trials were of comparable magnitude in both overweight groups (*p* = .32 and *p* = .14 for OW/OB-BED and OW/OB+BED, respectively).

The ANOVA across compound scores indicated no main effect of prime type, as would be predicted for a standardized variable. There was no main effect of the group factor (F [2, 89] = 1.15, *p* = .32, *η*_*p*_^2^ = .03), but an interaction of both factors (*F* [4, 178] = 2.44, *p* < .05, *η*_*p*_^2^ = .05). Post-hoc tests revealed that the interaction reflected the absence of group differences in the color prime condition, whereas both overweight groups had larger priming effects in office and food prime trials compared with the control group.

### Testing a change in priming effects across task blocks

The task comprised many trials in which a limited set of stimuli needed to be classified by button presses. As a consequence, associations between the stimuli and their respective task sets will become stronger across trials [85, 86]. If this leads to facilitated prime-based response activation, priming effects can be predicted to increase across trials. However, the training of response-interference resolution with trials could counter this effect. Therefore, we tested whether the magnitude of the priming effect changed from the first to the second half of the task, possibly moderating meaningful group differences. To this end, multivariate analyses of variance (MANOVA) were conducted with test-half (first vs. second) and group (OW/OB+BED vs. OW/OB-BED vs. NW) as factors, separately for RT and error scores. The MANOVA across RT indicated no main or interaction effect of the test half (all *p* > .09, *η*_*p*_^2^ < .07). Differently, the MANOVA across errors indicated a multivariate main effect of the test half (F [3, 86] = 3.86, *p* < .05; Wilks Λ = .88, *η*_*p*_^2^ = .12), but no interaction of test half with group (*F* [6, 172] = 0.88, *p* = .51; Wilks Λ = .94, *η*_*p*_^2^ = .03). The multivariate main effect of test-half was driven by an increase of the priming effect from the first to the second half only in office-prime trials (*p* < .001, *η*_*p*_^2^ = .12), whereas priming effects were comparable for both other prime categories (both *p* > .74, *η*_*p*_^2^ < .001).

### Correlations of effect scores

RT-based response priming in trials with a food-prime were correlated with the EDE-Q total score (*r* = .25, *p* < .05) and the weight concerns subscale (*r* = .27, *p* < .05). Also BMI was correlated with the priming effect in trials with a food-prime (*r* = .31, *p* < .01). None of the EDE-Q scales was correlated with error-based priming scores. Only BMI was found to be correlated with the error effect in trials with office-primes (*r* = .35, *p* < .01). In the compound scores, BMI was correlated with response-priming in trials with food (*r* = .28, *p* < .01) and with office primes (*r* = .29, *p* < .01). No other correlation was significant.

## Discussion

The aim of this study was to investigate early response inhibition, and to tear apart possible effects of overweight/obesity from that of BED. To this end a pictorial response-window priming paradigm [68, 69] was used that was recommended as a specific marker of early response inhibition [66]. The task was modified and comprised neutral and food related stimuli to test whether food-related response tendencies interfere more strongly than those elicited by neutral stimuli. Following the recommendation to tear apart effects of overweight/obesity from that of BED [61], three groups were compared: overweight/obese participants with BED (OW/OB+BED), overweight/obese participants without BED (OW/OB-BED), and normal weight controls (NW).

Confirming hypothesis (1), all groups revealed comparable response interference in case of neutral color primes. Confirming hypothesis (2), response interference was increased in trials with food-related stimuli in all OW/OB participants relative to NW controls. Disconfirming hypothesis (3), response interference was not increased in OW/OB participants with BED relative to OW/OB participants without BED. Confirming hypothesis (4), early response interference was found to be related with the severity of eating pathology.

Some details in the findings provoke questions that will briefly be discussed: Why were interference effects in color-prime trials generally small across all groups? As total priming effects (i.e. comprising both central priming and response priming), they could be predicted to exceed pure response priming effects. Further, color stimuli were faster (and more accurately) classified than both other stimulus categories. Consequently, larger interference could be predicted on the basis of the horse-race model [83, 84]. As all these accounts do not offer better explanations, the most plausible effect may be that response activation was simply stronger in food and office prime trials than in color prime trials. In part, this confirms our prediction that participants experience more difficulty to inhibit responses elicited by relevant primes.

But why did office utensils lead to comparable interference effects as food stimuli in the OW/OB groups? One possible explanation builds on two mechanisms: (1) Stimuli acquire associations with their respective task sets. Consequently, they can reactivate their task set in a bottom-up fashion [86]. (2) When classifying stimuli, the more salient category becomes a figure in front of the ground [87]. The food category should be generally more salient because of its evolutionary importance and its associations with reward [88, 89]. Participants will then solve the binary classification task by deciding whether the stimulus belongs to the salient category or not [87]. If these mechanisms hold in the current study, an office item could reactivate its task set, which would be to identify whether the current stimulus is food or not. Consequently, the response would be elicited in the context of food-related cognitions and would possibly receive a similar level of response activation or shielding. In support of this interpretation, the analyses across test halves indicated that error-based priming effects increased for office primes with task experience. This means, office items appear to become increasingly associated with their task set with task experience. Consequently, stronger prime-based task-set activation would lead to increased priming effects in office trials.

Why did priming effects appear in commission-error rates in office-prime trials, and why did they appear in response times in food-prime trials? This interesting effect may be accounted for by differences in classification times for stimulus categories in conjunction with the globally adjusted response window. Specifically, response times for office probes were generally faster than for food probes. Assuming response tendencies are also faster elicited for office primes, more commission errors can be expected following the horse-race model [83, 84]. Conversely, the RT effect can be better accounted for by stages models of interference control [65, 66]. As food-stimuli apparently took longer to be classified, a food-prime elicited response tendency may not have been processed up to the later stage of response execution when the probe is presented. Therefore, the likelihood of commission errors should not be increased. However, interference may still affect the response selection phase, and consequently response times. Therefore, RT-based priming effects may be purer indicators of early interference at response selection. Conversely, commission errors will only occur if inhibition additionally fails at the later stages.

Previous studies on impaired inhibition in overweight/obesity and BED have used laboratory tasks that tap late-stage response withholding and cancellation. Therefore, the findings obtained in the current study with a task that is more specific for early-stage response sampling rather complement previous findings. However, as the inhibitory functions operating at these stages are moderately overlapping, results will be briefly compared.

The present findings of increased interference of food-related information but not of neutral information in OW/OB individuals corresponds well with a stop-signal task study [54], in which BMI related cancellation impairments were only found for food-related stimuli but not for neutral stimuli. Similarly, BMI related impairments were found in a food-related go/no-go task [53], whereas no differences were observed between overweight and normal weight participants in a go/no-go study with neutral stimuli [47]. Differently from these bits of converging evidence, overweight individuals did not display more difficulties voluntarily suppressing an orienting response to food cues in an antisaccade task [50]. Possibly, the control of eye movements is less affected than that of showing an overt response. Additionally, there is evidence suggesting information processing is generally error prone in overweight/obese participants, also using neutral materials [55–58]. However, some of the tasks employed in these studies may not be considered pure measures of inhibition. Complementing behavioral evidence, functional imaging studies also found evidence that BMI is related with activity in neural regions associated with inhibition when participants were presented with food-stimuli. Depending on study characteristics, negative relationships may indicate impairment and positive relationships may indicate compensation (see [36] for an overview).

The present study yielded no evidence that early response interference control is impaired in OW/OB with BED relative to OW/OB without BED. Similarly, the absence of a difference in late-stage response cancellation between a BED group and weight-matched controls was shown in a SST with only neutral stimuli [60] and in multivariate study comparing extremely obese participants with and without BED [55]. Differently from these findings, response cancellation impairments were found in a SST in a BED group relative to weight-matched controls, particularly, when the response was elicited by a food-related stimulus [59]. Additionally, a BED group was found to commit generally more errors compared with obese participants without BED in a modified affective go/no-go task [58].

To summarize, the OW/OB associated deficits observed in this study can be reconciled well with previous findings of food-related inhibitory impairments found at later stages of information processing. The absence of a BED associated effect at early-stage response sampling stands in contrast with at least some of the studies reporting BED associated impairments at later stages of response withholding and cancellation. This suggests that food information interferes comparably in early-stage response selection in all OW/OB participants. However, additional impairments in BED to cancel a food-related approach tendency may contribute to the actually shown impulsive binging behavior in the latter group.

The results may have clinical implications. Given that food-related information interferes early in response selection, stimulus control might be indicated. However, given the omnipresence of palatable foods in today’s environment, stimulus control is hardly feasible. Therefore, a specific training to actively disengage attention from food items deserves being tested as a means of bias modification. For instance, an antisaccade training was suggested for BED [90]. Also training the currently used priming task may have similar effects, while targeting more specifically response selection. In fact, the most promising avenue may be to train simultaneously with multiple tasks in order to avoid idiosyncratic, task-specific optimization strategies [65].

A number of limitations shall be discussed that can compromise the interpretation of findings. Food consumption prior to the experimental session was neither controlled nor objectively tested in this study (e.g., with a glucose test). Compliance was only checked verbally by the laboratory assistant at study entry. We do not suspect that participants were generally dishonest or that groups differed systematically in the number of incompliant participants. However, it cannot be ruled out that some of the effects may have been moderated by previous food consumption or differential levels of state hunger. Therefore, future studies should better control for this possible limitation by offering a standardized meal prior to the experimental session.

Further, generalizability of the findings needs to be tested for the following reasons. First, as only women were included in this study, evidence of generalizability to overweight men with and without BED is still pending. This is important though, as obesity is equally and BED nearly equally prevalent in men. Second, the recruitment strategy applied in the current study deserves discussion. As announcements were made in diverse print media as well as radio and television, diverse participants may have received this information. However, those who actively responded and who wanted to participate in this effortful laboratory study might have been characterized by high levels of motivation and self-control. In this respect, participants of this study may be different from other OW/OB patients with or without BED. For instance, in a recent study it was shown that female BED patients were less persistent, less self-directed, and less cooperative than controls. Additionally, self-direction was reduced in OW/OB individuals without BED [91]. In spite of the finding that these self-control related factors are clearly separable from the low-level inhibitory control [62, 66] investigated in the current study, future studies should address whether the currently observed findings can be generalized to more heterogeneous groups of patients observed in typical clinical settings.

The stimuli used in this study were selected because they have been successfully employed in previous studies with OW/OB individuals and with BED patients. However, perceptual dissimilarities between stimulus categories could have affected response selection. For instance, pictures of office utensils tended to have more neutral color tones compared with food stimuli and color blobs. This perceptual distinctiveness could have contributed to their faster classification compared with food stimuli. In line with predictions from the horse-race model [83, 84], this would have contributed to the increased commission-error rates in office stimuli.

Finally, relationships of priming effects with eating pathology were only computed across the entire sample. It would have been interesting to compare the magnitude of the relationships within each group. However, this needs to be addressed in future studies given adequate sample sizes.

### Conclusion

This study has shown that early response interference is comparable in all groups as long as the presented stimuli are neutral. However, food-related stimuli cause larger response interference in all OW/OB individuals relative to NW controls. Conversely, OW/OB participants with BED appeared not to experience higher interference than other OW/OB participants. These results suggest that early-stage response selection is generally affected by food-related information in overweight/obesity. However, processes at this stage are not specifically impaired in BED.
